# Identification of proteins that specifically recognize and bind protofibrillar aggregates of amyloid-β

**DOI:** 10.1038/s41598-017-06377-8

**Published:** 2017-07-20

**Authors:** Elisabet Wahlberg, M. Mahafuzur Rahman, Hanna Lindberg, Elin Gunneriusson, Benjamin Schmuck, Christofer Lendel, Mats Sandgren, John Löfblom, Stefan Ståhl, Torleif Härd

**Affiliations:** 1 Department of Molecular Sciences, Swedish University of Agricultural Sciences (SLU), Uppsala BioCenter, Box 7015, SE-750 07 Uppsala, Sweden; 2 Affibody AB, Gunnar Asplunds Allé 24, SE-171 69 Solna, Sweden; 3 Division of Protein Technology, School of Biotechnology, Royal Institute of Technology (KTH), AlbaNova University Center, SE-106 91 Stockholm, Sweden; 4 Present Address: Department of Chemistry, School of Chemical Science and Engineering, Royal Institute of Technology (KTH), SE-100 44 Stockholm, Sweden

## Abstract

Protofibrils of the 42 amino acids long amyloid-β peptide are transient pre-fibrillar intermediates in the process of peptide aggregation into amyloid plaques and are thought to play a critical role in the pathology of Alzheimer’s disease. Hence, there is a need for research reagents and potential diagnostic reagents for detection and imaging of such aggregates. Here we describe an *in vitro* selection of Affibody molecules that bind to protofibrils of Aβ_42_cc, which is a stable engineered mimic of wild type Aβ_42_ protofibrils. Several binders were identified that bind Aβ_42_cc protofibrils with low nanomolar affinities, and which also recognize wild type Aβ_42_ protofibrils. Dimeric head-to-tail fusion proteins with subnanomolar binding affinities, and very slow dissociation off-rates, were also constructed. A mapping of the chemical properties of the side chains onto the Affibody scaffold surface reveals three distinct adjacent surface areas of positively charged surface, nonpolar surface and a polar surface, which presumably match a corresponding surface epitope on the protofibrils. The results demonstrate that the engineered Aβ_42_cc is a suitable antigen for directed evolution of affinity reagents with specificity for wild type Aβ_42_ protofibrils.

## Introduction

Aggregation of the amyloid-β peptide (Aβ) in the brain is a hallmark of Alzheimer’s disease^1^. The end-state of the aggregation is amyloid fibrils, which become deposited into senile plaques. However, Aβ aggregation involves a number of intermediate aggregation states, collectively called soluble oligomers, and evidence suggests direct causative links between soluble Aβ oligomers and synapse dysfunction^2, 3^. The aggregation path of the 42-residue Aβ_42_ peptide to amyloid fibrils is believed to involve the formation of pentameric or hexameric oligomers (paranuclei) that associate into larger protofibrils, which eventually undergo a structural interconversion into amyloid fibrils^4–6^. Please note that we refer to protofibrils as a class of rod-like pre-fibrillar aggregates that are distinct from amyloid fibrils^6^.

However, smaller dimeric or trimeric (low-n) oligomers^7^, and various aggregates that form in the presence of membranes or detergents, have also been described (and reviewed)^6^. In fact, much of the details surrounding oligomer formation and interconversion *in vivo* as well as *in vitro* remain elusive. New binding agents that specifically recognize intermediate aggregates, and which can be used to detect the presence of such aggregates, would represent valuable tools in the research and such agents might also be used within diagnostic or even therapeutic applications.

In this work, we describe the selection of Affibody molecules^8, 9^ that selectively recognize protofibrils of Aβ. There are several advantages and potential applications of *in vitro* selected binders. One apparent strength is that the state of target is under full control during the selection, which is not normally the case with IgG antibodies that are often generated by immunization. Another advantage, compared to IgG antibodies, is that Affibody molecules, in which the 58-residue small three-helix Z-domain is used as a scaffold for variation, are easily produced in large amounts in bacterial cultures or even by peptide synthesis. A third potential advantage relates to possible applications for therapy or brain imaging, as a smaller protein domain presumably will undergo a more rapid transfer through the blood-brain-barrier than large IgG antibodies. Finally, we wanted to address a more basic question in protein engineering: can an *in-vitro* selected protein binder be identified that can discriminate a certain protein aggregate from other aggregated and monomeric forms of the same peptide?

Protofibrils of wild type Aβ_42_ are instable and therefore not optimal as targets for binding protein selection using for instance phage display. However, we have previously engineered an Aβ_42_ cysteine variant (Aβ_42_ cc) that forms protofibrils, which do not convert into amyloid fibrils^10–12^. Briefly, in Aβ_42_ cc, alanine residues 21 and 30 are replaced with cysteine residues, allowing an intramolecular disulfide bond to form. The disulfide locks the peptide in a hairpin conformation, which is compatible with the conformation of Aβ_42_wt in protofibrils, but incompatible with the conformation observed in fibrils^13–15^. Aggregation of Aβ_42_ cc is therefore halted at the protofibril stage. In previous studies, size exclusion chromatography profiles, circular dichroism spectra and electron microscopy images showed a clear resemblance between the Aβ_42_cc protofibrils and what has previously been reported for Aβ_42_wt protofibrils^10^. We also demonstrated that the Aβ_42_cc protofibrils are recognized by a protofibril-selective antibody (mAb158). In a follow up study^11^, we characterized the biophysical properties of Aβ_42_cc protofibrils in detail and compared them with Aβ_42_wt protofibrils, using atomic force microscopy, analytical ultracentrifugation, nanoparticle tracking analysis, binding of the dye ANS, binding to oligomer-specific antibodies (A11 and OC) and a synaptotoxicity assay. In summary, none of the techniques revealed any significant differences between Aβ_42_cc protofibrils and Aβ_42_wt protofibrils, except the fact that Aβ_42_cc do not undergo structural conversion into amyloid fibrils.

Ideally, a comparison of the atomic structures of Aβ_42_cc and Aβ_42_wt protofibrils should be carried out to clarify the degree of structural similarity. However, structural biology of protein aggregates is extremely challenging because of sample heterogeneities. We have presented a structural model for the Aβ_42_cc protofibrils based on solid-state NMR data^12^, which is essentially different compared to the structure of Aβ_42_wt amyloid fibrils. Moreover, by a combination of magic angle spinning NMR and solution state NMR, we also showed that the N-terminal part of the peptide is disordered in the protofibrils^16^.

From previous studies, we can thus conclude that Aβ_42_ cc protofibrils are a good mimic of wild type Aβ_42_ protofibrils. Moreover, Aβ_42_ cc protofibrils are stable and thereby suitable as target for selection of binding proteins.

## Results

### Phage display selections

**Figure 1:**
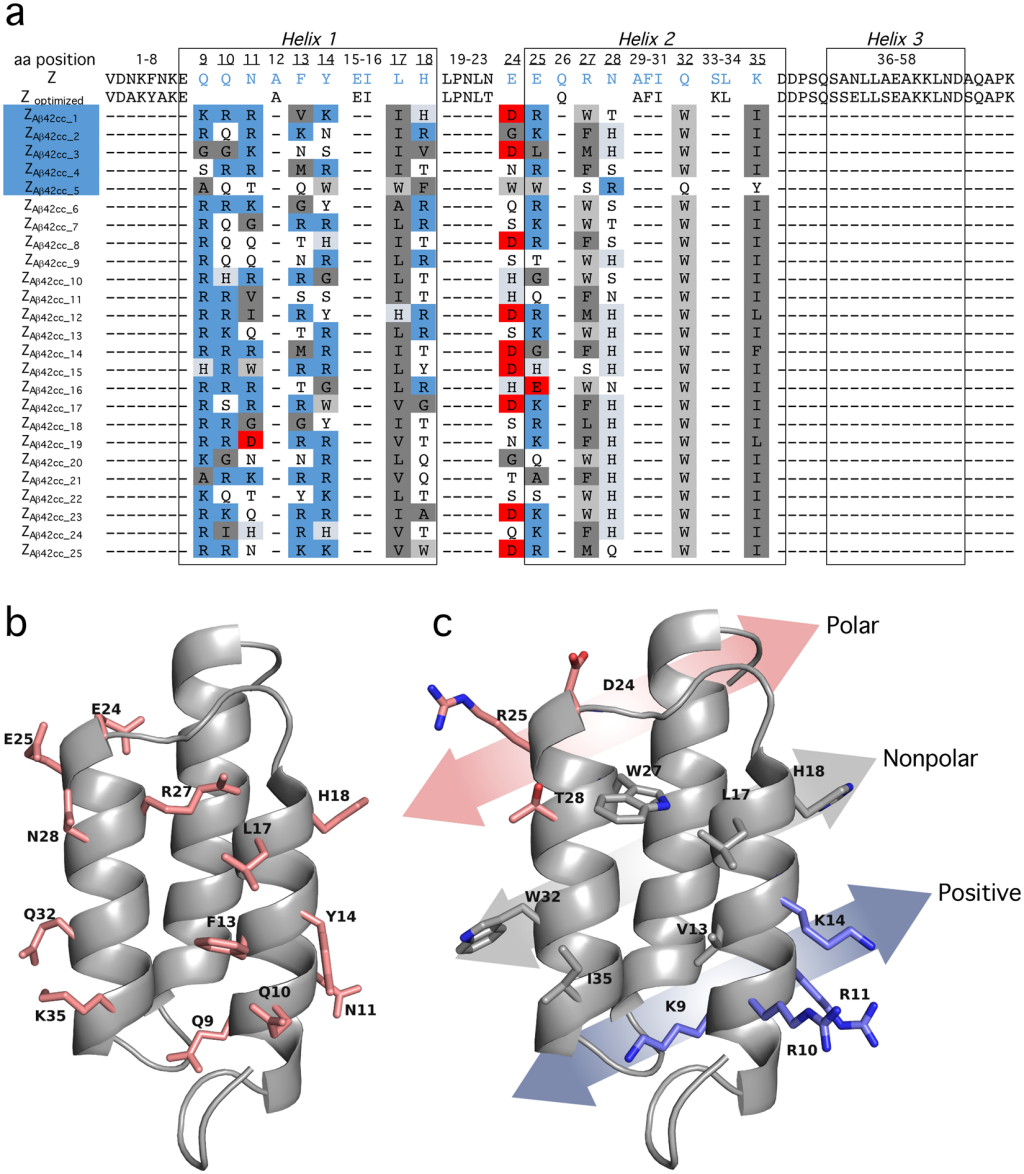
Sequence and cartoon representation of Affibody molecules selected as Aβ_42_cc protofibril binders. (**a**) Sequences of the top 25 selected Affibody molecules. The underlined amino acid positions are randomized in the phage display selection. The helical secondary structures are represented in boxes. The Affibody molecules chosen for further characterization are highlighted in blue. From 744 randomly picked clones, the sequence of Z_Aβ42cc_1_ was identified eight times, Z_Aβ42cc_2_ and Z_Aβ42cc_3_ one time, Z_Aβ42cc_4_ eight times and Z_Aβ42cc_5_ three times. (**b**) The original Z-domain scaffold. Side chains of residues that are subjected to variation in the phage library are indicated. (**c**) Representation of side chains that are mutated in binder Z_A__β__42cc_1_. The chemical properties of the side chains of the selected binders group into three chemically distinct regions of the surface, which identifies a ‘positive-nonpolar-polar’ recognition surface pattern.

Affibody molecules were selected using phage display with Aβ_42_ cc protofibrils as the target molecule. The protofibrils were biotinylated and attached to streptavidin coated magnetic beads. Binding proteins were selected from a phage library containing 1.4 × 10^10^ Affibody variants^17, 18^. A total of 13 surface-located amino acid residues in helices 1 and 2 of the Z-domain from *Staphylococcus aureus* protein A were randomized to construct the phage library^19^. The randomized positions can be found in Fig. 1a,b. This library was designed to exclude cysteines and should thus not allow for the selection of cysteine-containing Affibody molecules that can form dimers, such as the Aβ-monomer binding Affibody molecule that was selected and studied by us previously^17, 20^. The selection was carried out in two tracks, in which one involved a pre-selection against fibrils of Aβ_42_wt to remove fibril binders. Six rounds of selection and phage amplification were performed while gradually decreasing the target concentration from 2 µM (on a monomer basis) to 0.2 nM in the final round. Affibody variants in phage clones that remained after the fourth and final round were expressed as fusions to an albumin binding domain (ABD) and screened for protofibril binding performance using an enzyme linked immunosorbent assay (ELISA). In addition, it was vital for the study that none of the selected protofibril binders recognize mature amyloid fibrils. Therefore, the fibril binding properties were also assayed (see Supplementary Fig. S1). A total of 56 protofibril binders, identified in a single-point ELISA of 744 randomly selected Affibody variants, were ranked according to the apparent equilibrium dissociation constants (EC_50_ values; see Supplementary Fig. S2). It should be noted that these values are indicative of relative binding affinities in the ELISA format and not equilibrium dissociation constants. The sequences of 25 Affibody variants with the lowest ELISA EC_50_ values are shown in Fig. 1a. A comparison of sequence similarity in the form of a phylogenetic tree is shown in Supplementary Fig. S3.

Five strong Aβ_42_ cc protofibril binders, denoted Z_Aβ42cc_1_, Z_Aβ42cc_2_, Z_Aβ42cc_3_, Z_Aβ42cc_4_, and Z_Aβ42cc_5_, were selected for further characterization. These molecules were subcloned, expressed and purified. Three of these (Z_Aβ42cc_1_, Z_Aβ42cc_2_ and Z_Aβ42cc_4_) were selected because they appeared as strong binders in the ELISA. Z_Aβ42cc_3_ and Z_Aβ42cc_5_ were selected because their sequences differ from most other selected binders (Fig. 1a). At least Z_Aβ42cc_5_ most likely displays a different topology when binding to Aβ_42_cc protofibrils.

### Selectivity of binders to different Aβ aggregates

We then profiled the binding selectivity of the five selected binders (as mentioned above) to different Aβ aggregates using a second type of ELISA. This assay involved immobilization of Affibody-ABD fusions via an anti-ABD antibody followed by incubation with Aβ_42_cc protofibrils, Aβ_42_wt protofibrils (wt = wild type), Aβ_42_wt fibrils or Aβ_42_wt monomer. A non Aβ-binder Affibody molecule and PBS buffer were used as controls. The presence of bound Aβ remaining after washing was then detected using an HRP-conjugated antibody (6E10) that recognizes an N-terminal sequence of Aβ^21^ and which therefore most likely is insensitive to the Aβ aggregation state. A recent study suggests that the N-terminus of the peptide is disordered and more dynamic than the core structure of the protofibrils^16^.

In another assay, we used the antibody mAb1C3, a monoclonal mouse anti-Aβ IgG, which displays some specificity for Aβ_42_ protofibrils, compared to monomeric and low-molecular weight Aβ aggregates^21^. The Aβ_42_wt protofibrils used for this assay were made by either incubating Aβ_42_ monomer at 4 °C overnight or for 10 minutes at room temperature, followed by protofibril isolation by size exclusion chromatography^22^.

**Figure 2:**
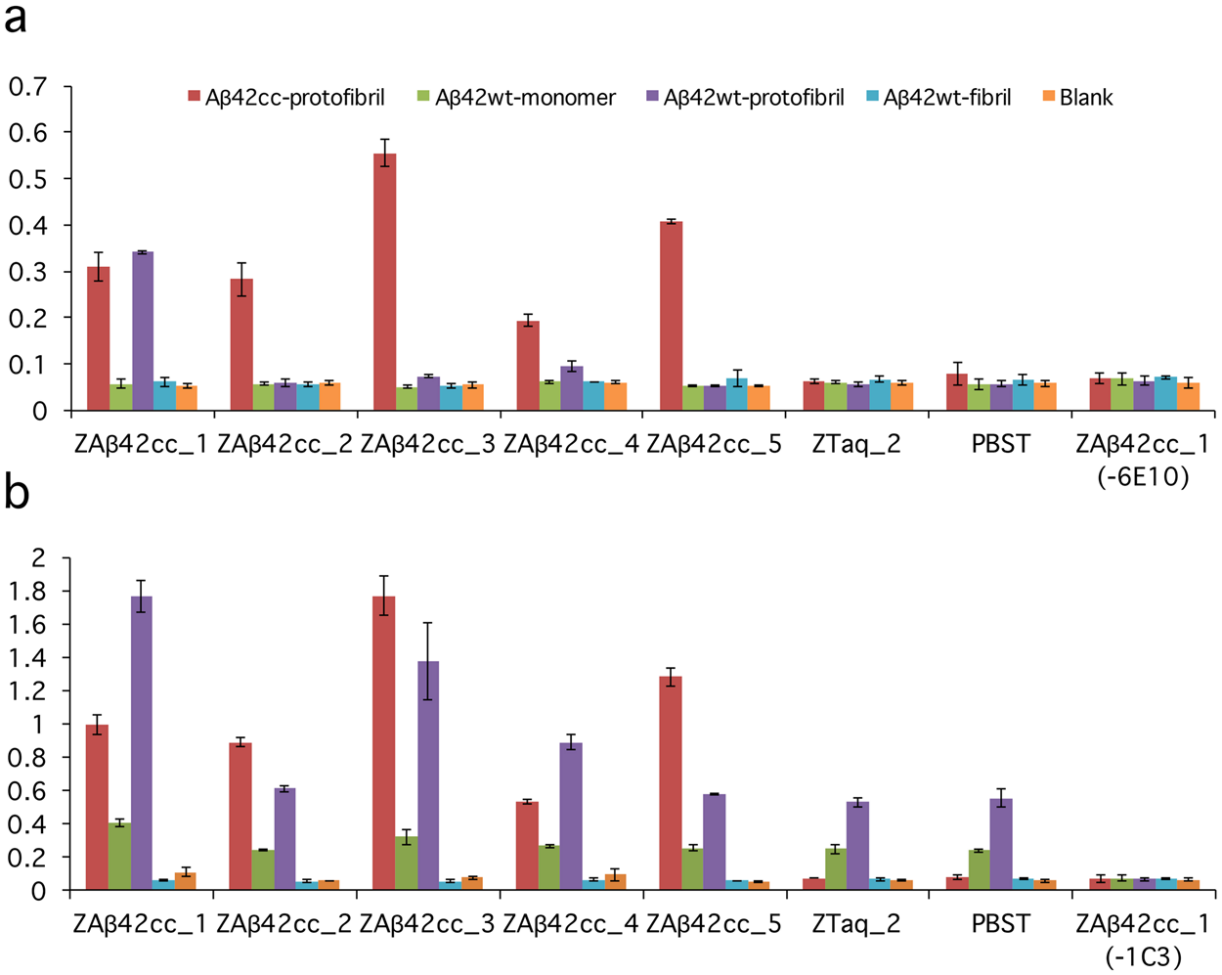
Binding profiling of five Affibody molecules to different Aβ aggregates, analyzed by ELISA. (**a**) Aβ_42_wt and Aβ_42_cc aggregates (50 nM assay concentration), bound to Affibody molecules, were detected by 6E10-HRP. 6E10^21^ recognizes the N-terminus of Aβ. As expected, all five Affibody molecules show binding to Aβ_42_cc protofibrils. The Affibody molecule Z_Aβ42cc_1_ also binds to Aβ_42_wt protofibrils. No binding could be observed to either wild type monomer or fibrils. (**b**) Same as in (**a**), except that the assay concentration of Aβ_42_ was 1 µM and mAb1C3 was used for detection (weakly specific for protofibrils^21^). The binding profile has the same pattern as in (**a**) for protofibrils (both wt and cc) and Aβ_42_wt fibrils, but with a higher background for Aβ_42_wt protofibrils. (**a**,**b**) Two controls involve replacing the Affibody molecule with either an irrelevant Affibody molecule (Z_Taq_2_) or by PBS-T. In a third control experiment PBS-T was added instead of Aβ-specific antibody (6E10 or mAb1C3). Values are means of duplicate experiments.

The five selected Affibody molecules all bound well to Aβ_42_cc protofibrils, as expected (Fig. 2). More important, three binders, and in particular Z_Aβ42cc_1_, also recognized Aβ_42_wt protofibrils, which was the intended outcome of the present selection. None of the five binders recognized monomer and fibrils of Aβ_42_wt.

**Figure 3:**
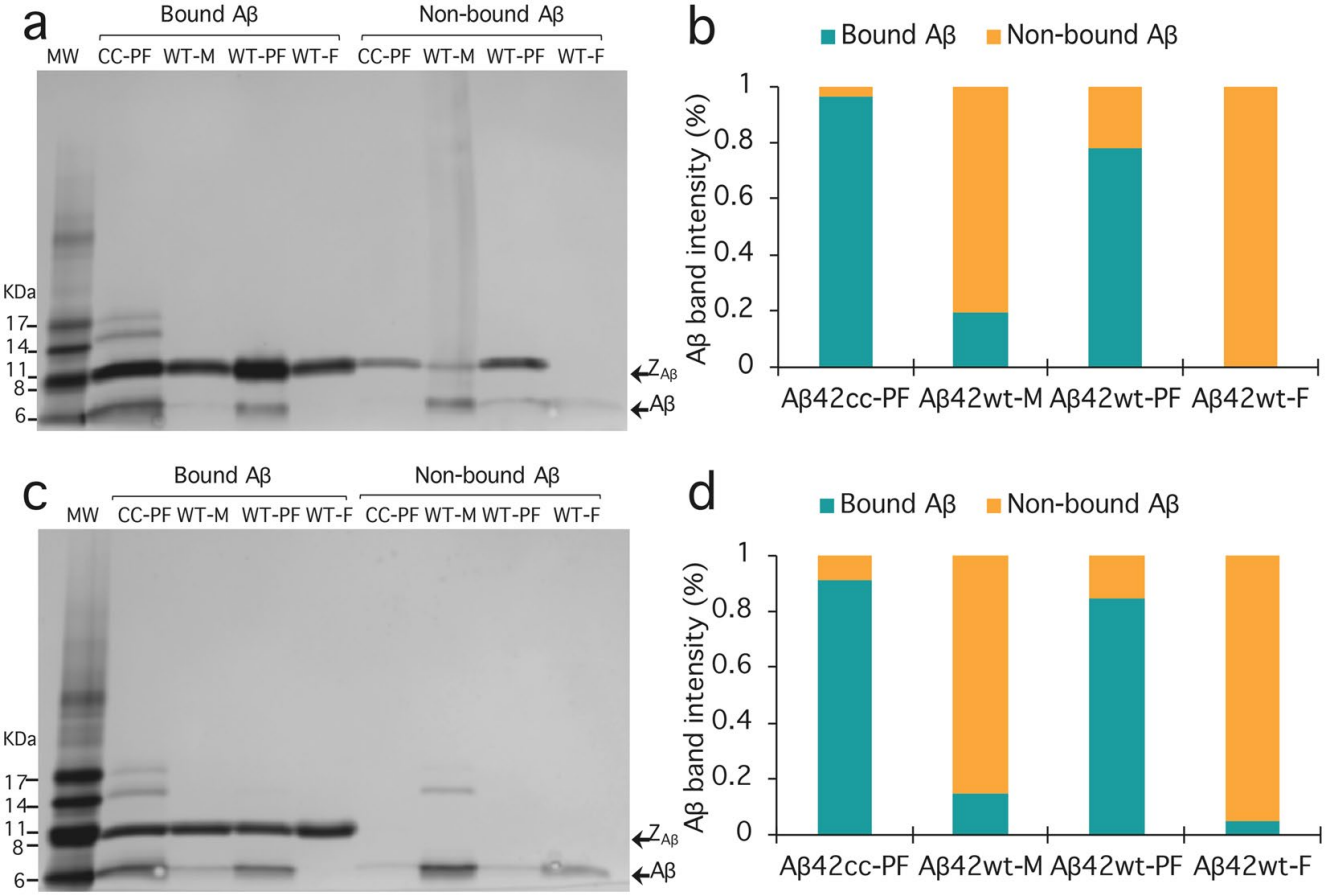
Binding of Affibody molecules to various Aβ aggregates and monomer in a batch mode affinity experiment. The SDS-PAGE analysis shows that Affibody Z_A__β__42cc_1_ (**a**) and Z_A__β__42cc_4_ (**c**) recognize protofibrillar aggregates of Aβ_42_cc and Aβ_42_wt (bound Aβ). Wild type monomer and fibrils did not bind to the Affibody molecules and were recovered in the supernatant (non-bound Aβ). The designations MW, CC-PF, WT-M, WT-PF and WT-F refer to molecular weight marker (GE Healthcare), Aβ_42_cc protofibrils, Aβ_42_wt monomer, Aβ_42_wt protofibrils and Aβ_42_wt fibrils, respectively. (**b**) and (**d**), Aβ band intensity for Z_A__β__42cc_1_ and Z_A__β__42cc_4_, respectively. The band intensities were obtained with the graphic program ImageJ^32^. The data was normalized with respect to each Aβ-species, i.e. the sum of the intensities for bands originating from one Aβ-species is equal to 100%.

We used an affinity based capturing approach to verify selective binding of Affibody molecules to protofibrils. For this experiment, we chose Z_Aβ42cc_1_ and Z_Aβ42cc_4_ since these two molecules, according to the selectivity profiling ELISA (Fig. 2a), recognize wild type protofibrils with an affinity that is close to that of Aβ_42_cc protofibrils. The Affibody molecules were recombinantly produced in *E*. *coli* and purified as described in the Methods section. They were immobilized on a nickel charged resin employing the His_6_ tag. The resin was briefly incubated with either Aβ_42_cc protofibrils, Aβ_42_wt protofibrils, Aβ_42_wt fibrils or Aβ_42_wt monomer. The resin was then pelleted and non-bound Aβ was collected in the supernatant, while bound Aβ was eluted together with Affibody molecules using a buffer containing imidazole. An SDS-PAGE analysis of the samples obtained from the affinity capture assay verifies that the two Affibody molecules Z_Aβ42cc_1_ and Z_Aβ42cc_4_ bind protofibrillar aggregates of Aβ_42_cc and Aβ_42_wt with similar selectivities, but do not recognize the monomer and fibrils of Aβ_42_wt (Fig. 3).

### Binding kinetics of Affibody molecules

**Figure 4:**
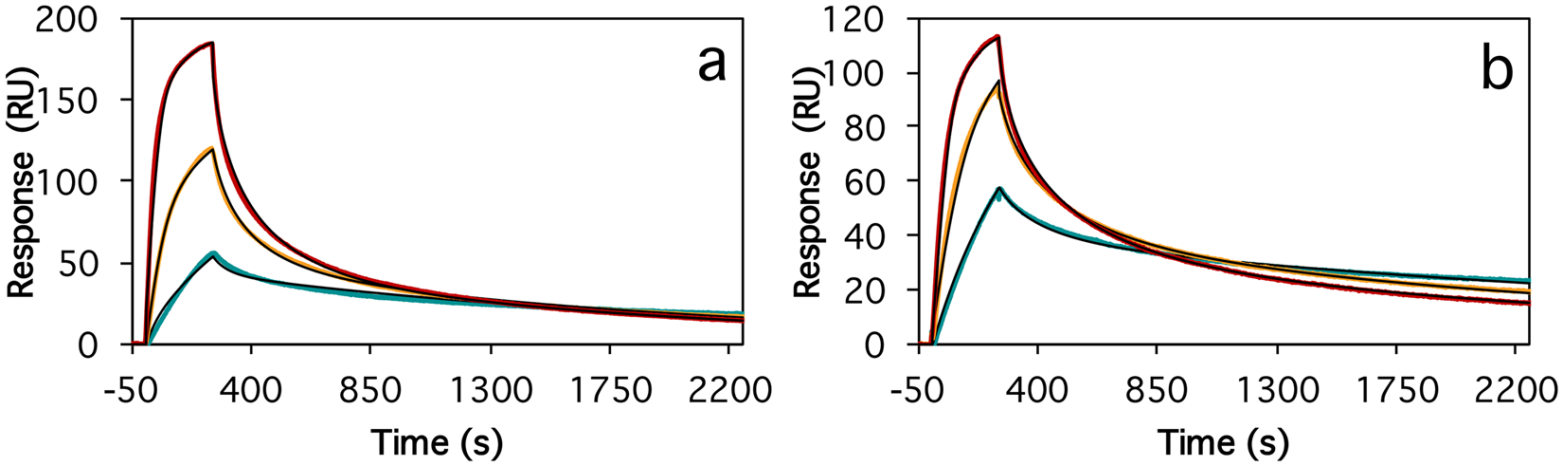
Association and dissociation kinetics for binding of two representative monomeric Affibody constructs to immobilized Aβ_42_cc protofibrils. Affibody concentrations are 10 (green), 20 (yellow) and 40 nM (red). The data was fitted to a heterogeneous ligand binding model with local maximum response (R_max_) values. The kinetics of the slow association and dissociation phases are *k*_*a*_ = 2.8 (±0.1) × 10^5^ s^−1^ M^−1^ and *k*_*a*_ = 2.5 (±0.1) × 10^5^ s^−1^ M^−1^ and *k*_*d*_ = 4.7 (±0.05) × 10^−4^ s^−1^ and *k*_*d*_ = 6.2 (±0.05) × 10^−4^ s^−1^ for Z_Aβ42cc_1_ (**a**) and Z_Aβ42cc_4_ (**b**), respectively, which correspond to dissociation constants for the stronger binding site of *K*_*D*_ = 1.6 (±0.1) and *K*_*D*_ = 2.5 (±0.2) nM, respectively.

Next, we used surface plasmon resonance (SPR) to study the kinetics for association and dissociation of the selected Affibody molecules to Aβ_42_cc protofibrils. For this we immobilized Aβ_42_cc protofibrils on a Biacore CM5 sensor chip using amine coupling chemistry. Affibody binding kinetics to this surface (Fig. 4 and Supplementary Table S1) was in all cases characterized by non-exponential association and non-exponential and saturation dependent dissociation. Collected data did fit well to a global heterogeneous binding model with two binding sites, if local maximum response (R_max_ value) was assumed. The monomeric Affibody molecules, also in ABD-fused format, all bound with similar affinities and kinetics with average dissociation constants for the high-affinity sites of *k*_*d*_ = 5 (±2) × 10^−4^ s^−1^ and *K*_*D*_ = 1.7 (±0.6) nM. A non Aβ-binding Affibody molecule (Z_Taq_1_) and a Z_Taq_1_-ABD fusion protein were used as controls. Of these, Z_Taq_1_ showed no binding, and Z_Taq_1_-ABD showed very weak binding to Aβ_42_cc protofibrils (results not shown).

### Binding of dimeric Affibody constructs

**Figure 5:**
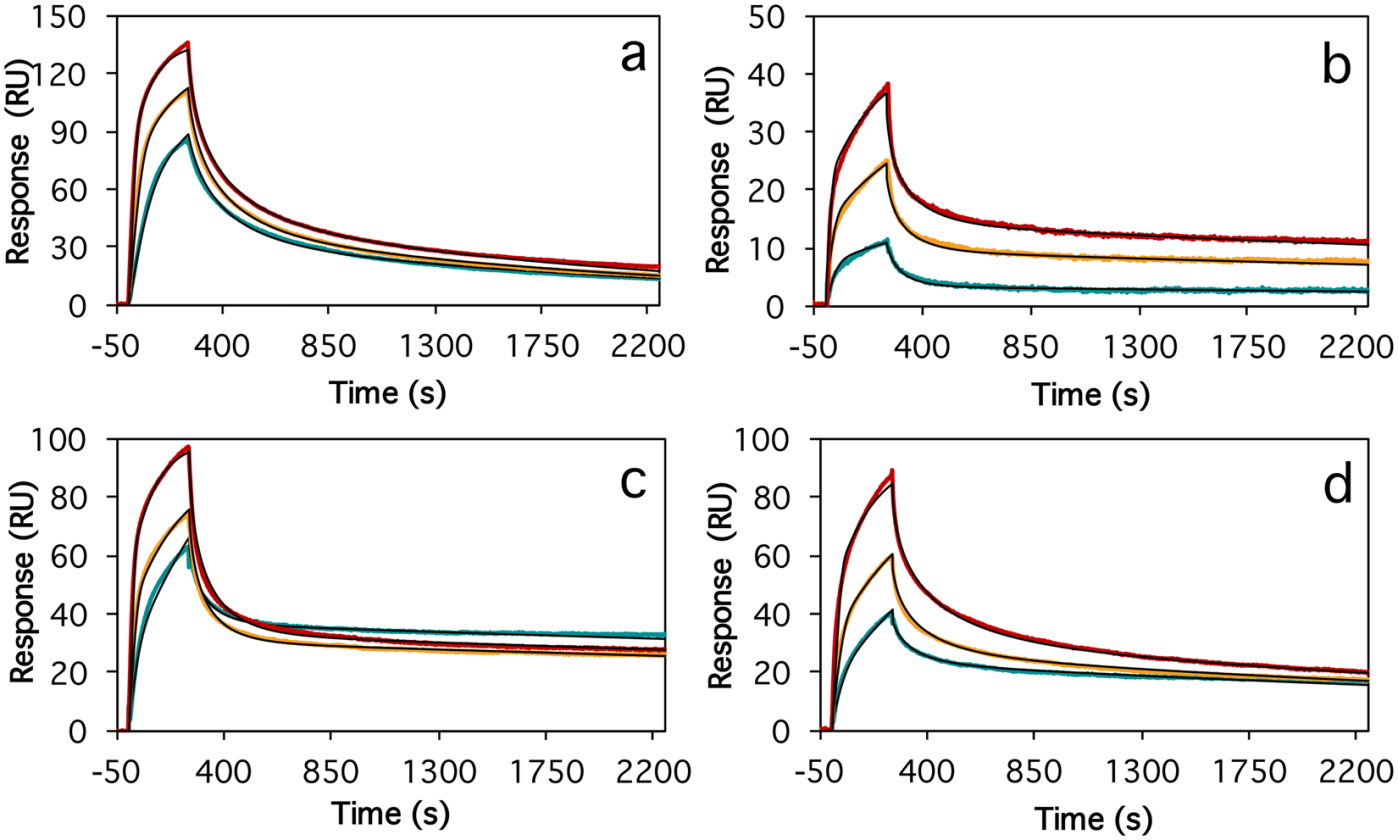
Association and dissociation kinetics for binding of some of the head-to-tail Affibody dimers to immobilized Aβ_42_cc protofibrils. Data were fit to a heterogeneous ligand binding model with local maximum response (R_max_) values. (**a**) Z_Aβ42cc_1_-(GGGGS)- Z_Aβ42cc_1_-ABD with Affibody dimer concentrations of 10 (green), 20 (yellow) and 40 nM (red). The kinetics of the slow association and dissociation phases are *k*_*a*_ = 3.1 (±0.1) × 10^5^ s^−1^ M^−1^ and *k*_*d*_ = 5.2 (±0.03) × 10^−4^ s^−1^ corresponding to a dissociation constant for the stronger binding site of *K*_*D*_ = 1.7 (±0.1) nM. (**b**) Z_Aβ42cc_1_-(GGGGS)_4_- Z_Aβ42cc_1_-ABD; 10, 20 and 40 nM; *k*_*a*_ = 1.4 (±0.1) × 10^5^ s^−1^ M^−1^, *k*_*d*_ = 1.6 (±0.01) × 10^−4^ s^−1^, *K*_*D*_ = 1.1 (±0.1) nM. (**c**) Z_Aβ42cc_4_- Z_Aβ42cc_4_ (no linker); 7.8, 15.5 and 31.2 nM; *k*_*a*_ = 3.4 (±0.1) × 10^5^ s^−1^ M^−1^, *k*_*d*_ = 9.1 (±0.01) × 10^−5^ s^−1^, *K*_*D*_ = 0.27 (±0.01) nM. (**d**) Z_Aβ42cc_4_-(GGGS)_4_- Z_Aβ42cc_4_; 7.8, 15.5 and 31.2 nM; *k*_*a*_ = 2.2 (±0.3) × 10^5^ s^−1^ M^−1^, *k*_*d*_ = 2.5 (±0.01) × 10^−4^ s^−1^, *K*_*D*_ = 1.1 (±0.05) nM.

Finally, we investigated if the affinity of the selected Affibody molecules could be improved even further by linking the binders into head-to-tail dimers. For this, we constructed a number of dimeric fusion proteins with (GGGS)_n_ or (GGGGS)_n_ linkers of variable lengths and studied their binding to Aβ_42_cc protofibrils by SPR (Fig. 5 and Supplementary Table S1). In total, we studied 14 dimeric constructs of four Affibody molecules with or without ABD-fusions. All dimers, except one for which the slow binding off-rate could not be determined, bound with affinities that are either equal to those of the corresponding monomers, or up to one order of magnitude stronger compared to the monomers. There was, in two of four cases, an apparent effect of linker length on binding affinity. For instance, dimeric Z_Aβ42cc_4_ without linker (Z_Aβ42cc_4_–Z_Aβ42cc_4_) bound with a *K*_*D*_ = 0.26 nM, intermediate length linkers showed a *K*_*D*_ = 0.6 to 0.7 nM, and the Z_Aβ42cc_4_ dimer with the longest linker (Z_Aβ42cc_4_–(GGGS)_4_–Z_Aβ42cc_4_) appeared to bind somewhat weaker (*K*_*D*_ = 1.1 nM). This trend was however not common to the whole dimer binding data set.

## Discussion

Research in the field of Alzheimer’s disease is in need of new good methods to detect and distinguish various forms of aggregated amyloid-β. Conformation specific monoclonal antibodies have previously been developed for this purpose^21, 23, 24^. Here, we explore alternatives to IgG antibodies by the *in vitro* selection of Affibody molecules binding to protofibrillar aggregates of Aβ_42_. Such binders would potentially have a number of technological advantages, as compared to using antibodies, in applications including tissue imaging, use of PET ligands, diagnostic biomarker profiling and basic research^8^.

We used protofibrils formed by Aβ_42_cc as the target for binding protein selection. Aβ_42_cc contains an intramolecular disulfide that locks the peptide in a hairpin conformation that is incompatible with the conformation of Aβ in amyloid fibrils. Aβ_42_cc aggregation is therefore halted at the protofibrillar state. We have in previous studies shown that Aβ_42_cc protofibrils are good mimics of protofibrils formed by wild type Aβ_42_^10, 11^.

However, it was essential that the selected binding proteins also recognized wild type Aβ_42_ aggregates. Therefore, we performed a profiling assay in an ELISA format to assess this aspect (Fig. 2). The results with conformation independent 6E10 antibody detection indicate that one, or possibly three, of the five tested Affibody molecules bound wild type protofibrils with an affinity that, at least in the case of Z_Aβ42cc_1_, was close to that for binding of Aβ_42_cc. Detection using the weakly protofibril conformation-selective mAb1C3 monoclonal antibody indicated that wild type protofibrils remained bound to Z_Aβ42cc_1_, Z_Aβ42cc_3_ and possibly Z_Aβ42cc_4_ at the time of detection. It cannot be excluded that all Affibody molecules indeed recognize wild type protofibrillar aggregates with high affinity. This is because of the rapid interconversion of wild type protofibrils into mature fibrils that occurs within hours^25^. Hence, with the incubation times for Affibody and antibody binding in the ELISA experiment results, shown in Fig. 2 (one hour each at room temperature), wild type protofibrils, except those most strongly bound, might have converted into fibrils, which would have been washed off before detection. Hence, the ELISA in Fig. 2 is a very stringent assay and repeating these experiments with shorter incubation times might reveal that all selected binders recognize wild type protofibrillar aggregates.

We employed an affinity capture assay to confirm the aggregate selectivity of the Affibody binders. Shorter incubation times were chosen here in order to minimize the intricacy originating from the maturation of the protofibrils to fibrils^25^, and thus improve the resolution for detection of selective binding. The results confirm selective binding of Z_A__β__42cc_1_ and Z_A__β__42cc_4_ to protofibrils. However, in contrast to the ELISA selectivity profiling, where the selectivity of Z_Aβ42cc_1_ to Aβ_42_cc protofibrils was higher than Z_Aβ42cc_4_, both these Affibody molecules bind Aβ_42_cc protofibrils to a similar extent. Interestingly, the results suggest that these two Affibody molecules also recognized Aβ_42_wt protofibrils and Aβ_42_cc protofibrils with similar selectivity. A possible explanation for this “selectivity improvement” compared to the ELISA profiling is the reduced incubation time, which would be in line with the intention of this experiment.

The SPR data indicated that the best selected Affibody molecules interacted with Aβ_42_cc with slow off-rates in the order of *k*_*d*_ = 1 to 7 × 10^−4^ s^−1^, and equilibrium dissociation constants in the range *K*_*D*_ = 1 to 3 nM. The (total) concentrations of soluble Aβ_42_ in body fluids of healthy humans range from *ca*. 200 pM in the cerebrospinal fluid to *ca*. 20 pM in blood plasma. The concentration of Aβ_42_ protofibrils in brains of patients with Alzheimer’s disease has not been determined, but it should be lower, and perhaps much lower, than the total soluble Aβ_42_ concentration. Hence, it would be desirable to develop even stronger binding Affibody molecules to ensure the detection of protofibrillar species. To reach this, we explored the possibility to link Affibody molecules into dimers to achieve even slower off-rates and thereby higher affinity. The rational for this strategy was that protofibrillar protein aggregates should have a modular morphology in which Affibody binding sites are repeated in a regular manner. Hence, simultaneous engagement of two closely located sites by a single molecule (a dimer) should increase the affinity. In fact, we recently demonstrated that Affibody dimers can be improved 1000-fold compared to monomers as binders of a protein containing multiple similar binding surfaces^26^.

With dimers of Affibody molecules we also achieved slower off-rates, as expected, and in some cases also significantly higher affinities (K_D_ of around 300 pM) compared to monomer binding, but the affinity gain was not quite as great as one would have hoped based on earlier studies. We do not completely understand why higher affinities are not achieved and further studies, including structural comparisons of monomeric and dimeric constructs, are necessary to address this issue.

Sequences of selected Affibody molecules are, with a few exceptions, homologous (Fig. 1a). In fact, 23 of 25 of the listed sequences in Fig. 1a appear to reflect the same binding surface (Fig. 1c). One of these sequence (Z_Aβ42cc_3_) appears remotely homologous, and one (Z_Aβ42cc_5_) appears to reflect a different binding surface. The 23 homologous sequences are characterized by the selection of positively charged residues, and in particular arginines, at positions 9, 10, 11, and to large extent also at positions 13, 14 and 25. Similarly, nonpolar side chains are selected at positions 17, 27, 32 and 35. Interestingly, tryptophan occurs at position 32 in all conserved sequences. The homology at positions 24 and 28 are predominantly polar, perhaps with a tendency for negative and positive charge, respectively. Position 18 does not clearly appear to have been subjected to any selection pressure.

The chemical properties of selected side chains define three chemically distinct regions of the Z-domain scaffold surface as illustrated in Fig. 1c. Residues 9, 10, 11, 13, 14, which are rich in basic residues, form an area of strong positive electrostatic potential. Adjacent to this surface is a non-polar surface (residues 17, 27 and 35) with a nonpolar and bulky tryptophan side chain (residue 32) at the edge. Finally, the nonpolar surface is flanked by a polar surface. The surface pattern positive-nonpolar-polar presumably matches the binding epitope on Aβ_42_cc protofibrils. In fact, the structural model of a hexameric protofibril building block that recently was derived by our group, using solid-state nuclear magnetic resonance data and Rosetta modeling^12^, indeed contains surfaces that appear to match the selected Affibody molecules. However, further structural studies are needed to confirm the binding site at the protofibrils.

To conclude, amyloid peptides are difficult to use as antigens in directed evolution. These peptides rapidly form different forms of aggregates and the antigen hence tends to be a mix of different molecular species, hampering reproducibility, specificity for the intended form and success rate. Here, we show that using the engineered Aβ_42_cc, we prevent formation of higher order aggregates and trap the peptide in the protofibril state to obtain a more defined antigen for successful selection of specific binders, which importantly also recognise Aβ_42_wt protofibrils. We believe that the approach could facilitate future efforts on development of affinity reagents (including monoclonal antibodies) for specific forms of Aβ aggregates and in particular for Aβ protofibrils.

## Methods

### Aβ production and preparation of aggregates

Aβ_42_cc and Aβ_42_wt were produced by co-expression with the Z_A__β__3_ Affibody molecule and purified as described previously^10, 11, 27^. Aβ peptide was separated from the Z_A__β__3_ by denaturation in 7 M guanidinium hydrochloride followed by immobilized metal ion affinity chromatography (IMAC) under denaturating conditions.

Aβ_42_cc protofibrils were obtained by overnight dialysis of monomeric fractions against 20 mM Na-phosphate, pH 7.4, 50 mM NaCl, 1 mM EDTA at room temperature. The dialysis was continued for another 7 h in the same buffer without EDTA, followed by heating to 60 °C for 10 min.

Protofibrils of Aβ_42_wt were freshly prepared for each experiment, essentially as described in ref. 25, and used immediately. Aβ_42_wt monomer was diluted to a concentration of 100 µM and the pH was adjusted to 7.4 (with 1 M HCl). Protofibrils were allowed to form either overnight at 4 °C or for 10 minutes at room temperature. The protofibrils fraction (elution volume, V_e_ = 8 mL) was isolated from monomeric fraction (V_e_ = 13 mL) by size exclusion chromatography (SEC) using a Superdex 75 10/300 GL column (GE Healthcare) with 20 mM NaPi, pH 7.4, 150 mM NaCl buffer.

To assemble Aβ_42_wt fibrils, the monomer was centrifuged to pellet any existing insoluble aggregate and the soluble fraction was diluted to 25 µM with phosphate buffered saline (PBS: 2.68 mM KCl, 1.47 mM KH_2_PO_4_, 137 mM NaCl, 8.1 mM Na_2_HPO_4_, pH 7.4) and incubated at 28 °C with 110 rpm shaking for 80 h^22^. The fibrils were spun down and washed twice with PBS.

### Phage display selections

A combinatorial phage library of the Z domain, with randomized positions 9, 10, 11, 13, 14, 17, 18, 24, 25, 27, 28, 32 and 35, was prepared as described in^9, 17, 19, 28^. Aβ_42_cc protofibrils and Aβ_42_wt fibrils were biotinylated as described in ref. 18, and the unreacted biotin was removed by SEC on a Superdex 200 10/300 GL column (GE Healthcare). The selection and amplification were performed in PBS-T (0.1% Tween20 added to PBS) at room temperature as described previously^26^. Prior to the first cycle of protofibril biopanning, fibril binders were removed from the library by incubation of phages with pre-adsorbed streptavidin beads. The remaining phage particles in the supernatant were panned for 1.5 − 2.0 h against Aβ_42_cc protofibrils in six cycles. The concentration of Aβ_42_cc protofibrils was 2 µM (monomer subunit concentration) in the first cycle, and decreased five times in subsequent selection rounds. In the last cycle, the library was panned in three parallel tracks using a 4–0.2 nM target concentration. DNA sequencing of the generated binders was performed as previously described^26^.

### Screening ELISA

Phages from randomly selected clones after the fourth and sixth selection rounds were produced and screened for Aβ_42_cc protofibril binding activity by an ELISA. The expression was performed directly from the phagemid vector, which yielded the Affibody variants as albumin binding domain (ABD) fusion proteins that became secreted in the *E*. *coli* periplasm, as described previously^18^. For ELISA, 50 μL periplasmic fraction was transferred to Costar high binding half area 96 well plates (Corning), previously coated with 2 µg/mL of a goat anti-ABD IgG HP001, and blocked with PBSC (0.5% Casein (Sigma) in PBS), for 1.5 h incubation. The plates were washed four times with PBS-T, prior to addition of 42 nM biotinylated Aβ_42_cc protofibrils per well and incubated for 1 h. After washing the wells four times, streptavidin-HRP (Dako) diluted 1:30,000 in PBSC was added to the wells and incubated for 1 h. TMB substrates A and B were mixed 1:1 and added to washed wells and incubated for 7 min according to the manufacturer’s instructions (ImmunoPure TMB Substrate Kit; Thermo Scientific). Stop solution (2 M H_2_SO_4_) was added and the absorbance at 450 nm was measured in an ELISA reader (Victor 3, Perkin Elmer).

### Binding profiling to different Aβ aggregates

The five Affibody variants (Fig.1,a) as ABD-fusion proteins were analyzed for ability to bind different aggregated forms of Aβ_42_cc and Aβ_42_wt using an ELISA assay at room temperature. Briefly, 50 μL periplasmic fraction was transferred to Costar high binding half area 96 well plates (Corning), previously coated with 2 µg/mL of HP001 and blocked with 0.5% Casein (Sigma) in PBS for 1.5 h incubation. The plates were washed four times with PBS-T prior to addition of 50 nM or 1 µM Aβ_42_ peptide solutions and incubation for 1 h. After washing, plates were incubated with mAb1C3, monoclonal mouse anti-Aβ IgG, (2 µg/mL)^21^, or mAb(6E10)-HRP, mouse anti-Aβ IgG1 (33 ng/mL) (SIG-39345, BioSite) for 1 h. Prior to washing, wells with mAb1C3, were incubated with an anti-mouse-HRP antibody (G21040, Invitrogen) for 1 h. TMB substrates A and B were mixed at a ratio of 1:1, and added to washed wells and incubated for 5 to 30 min according to the manufacturer’s instructions (ImmunoPure TMB Substrate Kit; Thermo Scientific). Stop solution (2 M H_2_SO_4_) was added to the wells, and the absorbance was measured at 450 nm in an ELISA plate reader (Victor 3, Perkin Elmer). An Affibody molecule specific for Taq-polymerase (Z_Taq_2_) was used as negative control.

The selectivity of binders to different Aβ aggregates was also tested using Affibody molecules as ligands in a batch mode experiment. For this experiment, the Affibody molecules were essentially produced as described below. Z_Aβ42cc_1_ or Z_Aβ42cc_4_ (65 µg) were immobilized on 75 µL Ni^2+^ charged IMAC Sepharose 6 fast flow (GE Healthcare) by incubation for 20 min at room temperature. The beads were pelleted by centrifugation and washed with buffer A (10 mM Tris-HCl, pH 7.4) to remove unbound Affibody molecules. In total 1.7 µg of Aβ_42_cc protofibrils, Aβ_42_ protofibrils, Aβ_42_ fibrils and Aβ_42_ monomer were separately incubated with Affibody bound IMAC Sepharose for 5 min at room temperature. Aβ_42_wt protofibrils were freshly prepared by SEC as explained above and used immediately. The IMAC-Affibody-Aβ complex was pelleted and the supernatant was transferred into new tube. The complex was washed once with buffer A. The Affibody-bound Aβ was eluted with 20 mM NaPi pH 7.4, 50 mM NaCl, 300 mM imidazole. Eluate and supernatant were analyzed using SDS-PAGE. The samples were prepared in SDS-PAGE sample buffer (Bio-Rad) containing 15 mM TCEP in final concentration (for fibril sample 8 M urea was included). Samples were heated to 90 °C for 5 min prior to loading on Criterion precast 4–20% gradient gels (Bio-Rad). Protein bands were visualized by silver staining^29^. The gels were scanned using Gel Doc EZ Imager (Bio-Rad).

### Affibody molecule production

The Affibody molecules selected for further characterization were produced with N-terminal His_6_ tags or C-terminal ABD fusion molecules for purification.

For N-terminal His_6_ tag constructs, the Affibody molecules were cloned into pET28b(+) expression vectors, yielding the final constructs GSSHHHHHHLQ[Z_Aβ42cc_X_]VD, where Z_Aβ42cc_X_ denotes the selected Affibody molecule. *E*. *coli* BL21STAR (DE3) cells (Invitrogen) were transformed with the expression plasmids and cultivated at 37 °C in TB medium (50 µg/L kanamycin). At an OD_600nm_ of approximately 1, protein expression was induced by addition of IPTG to a final concentration of 0.2 mM. The temperature was lowered to 16 °C and the culture was incubated for 18 h prior harvest. The protein purification was done by an IMAC purification using a 5 mL HiTrap Chelating HP column (GE Healthcare) with 20 mM Tris-HCl, pH 8.0, 0.5 M NaCl as running buffer, and 20 mM Tris-HCl, pH 8.0, 0.5 M NaCl, 400 mM imidazole for elution of bound protein. The IMAC purification was followed by a SEC purification using a Superdex 75 10/300 GL column (GE Healthcare), with 20 mM NaPi, pH 7.4, 150 mM NaCl as running buffer.

The genes for the Affibody molecules with a C-terminal ABD_035_ tag^30^ were cloned into the expression vector pET26b(+). The obtained constructs were [Z_Aβ42cc_X_]-ABD_035_, where Z_Aβ42cc_X_ denotes the selected Affibody molecule. The encoded proteins were essentially expressed as described above, however using a TSB + Y as cultivation medium and 1 mM IPTG for induction of the protein expression. The Affibody molecules were purified by affinity chromatography on a 2 mL anti-ABD agarose column (Affibody AB) using tris-buffered saline (TST: 25 mM Tris-HCl, 200 mM NaCl, 1 mM EDTA, 0.5% (w/v) Tween 20, pH 8.0) as running buffer, 5 mM NH_4_Ac (pH 5.5) for washing and 0.5 M HAc (pH 2.8) for elution. The eluted proteins were buffer-exchanged to PBS on a NAP-10 columns (GE Healthcare).

Head-to-tail dimeric constructs were designed with (GGGS)_n_ or (GGGGS)_n_ linkers, and with a N-terminal His_6_ tags or C-terminal ABD-tags for purification, respectively. The proteins obtained were GSSHHHHHHLQ[[Z_Aβ42cc_X_]-(GGGS)_n_-[Z_Aβ42cc_X_]], or [[Z_Aβ42cc_X_]-(GGGGS)_n_-[Z_Aβ42cc_X_]]-ABD035, where Z_Aβ42cc_X_ denotes the selected Affibody sequence. Dimeric Affibody molecules were expressed and purified as described for the monomer. The molecular weight of the purified proteins was verified by LC/MS (Agilent Technologies 6520 ESI-Q-TOF).

### Surface plasmon resonance analysis

Surface plasmon resonance (SPR) studies were performed on a Biacore X100 instrument (GE Healthcare). The Aβ_42_cc protofibrils were immobilized onto a Biacore CM5-sensor chip (GE Healthcare), as described previously^31^.

Five or six concentrations of each analyte were prepared in HBS-EP (10 mM HEPES, 150 mM NaCl, 3 mM ETDA, 0.005% Tween-20, pH 7.4) and injected over the immobilized chip surface for 250 s to record analyte binding to the surface. Dissociation was observed for 2,000 s in running buffer. The sensor surface was regenerated after each injection with 20 mM NaOH with 90 s contact times. All experiments were carried out at 25 °C with a flow rate of 10 μL/min.

SPR data sets were analyzed using Biacore X100 Evaluation 2.0.1 software and curve fitting was performed with a heterogeneous binding site model using global kinetic fitting, but with local adjustment of the parameter R_max_.

## Electronic supplementary material

Supplementary information
