# Supplementary material for: Identification of proteins that specifically recognize and bind protofibrillar aggregates of amyloid-β
Source: Sci Rep. 2017 Jul 20;7:5949. doi: 10.1038/s41598-017-06377-8 (PMC5519597; doi:10.1038/s41598-017-06377-8)
Supplement: Supplementary file 1 — Supplementary information [file 41598_2017_6377_MOESM1_ESM.pdf]

Supplementary information

Identification of proteins that specifically recognize and bind protofibrillar aggregates of amyloid-β

Elisabet Wahlberg[§,1,2], M. Mahafuzur Rahman[§,1], Hanna Lindberg[3], Elin Gunneriusson[2], Benjamin Schmuck[1], Christofer Lendel[‡,1], Mats Sandgren[1], John Löfblom[3], Stefan Ståhl[3], and Torleif Härd[*,1]

[1]Department of Molecular Sciences, Swedish University of Agricultural Sciences (SLU), Uppsala BioCenter, Box 7015, SE-750 07 Uppsala, Sweden

[2]Affibody AB, Gunnar Asplunds Allé 24, SE-171 69 Solna, Sweden

[3]Division of Protein Technology, School of Biotechnology, Royal Institute of Technology (KTH), AlbaNova University Center, SE-106 91 Stockholm, Sweden

[*]To whom correspondence should be addressed. E-mail: torleif.hard@slu.se

[§]E.W. and M.M.R. contributed equally to this work

[‡]Present address: Department of Chemistry, School of Chemical Science and Engineering, Royal Institute of Technology (KTH), SE-100 44 Stockholm, Sweden

Contents

- **Figure S1** – Single-point ELISA screen
- **Figure S2** – EC_{50}-ELISA of the 25 best Affibody molecules
- **Figure S3** – Sequence similarity comparison (phylogenetic tree) of 25 selected Affibody molecules
- **Table S1** – Fits of surface plasmon resonance data to kinetics model

a.

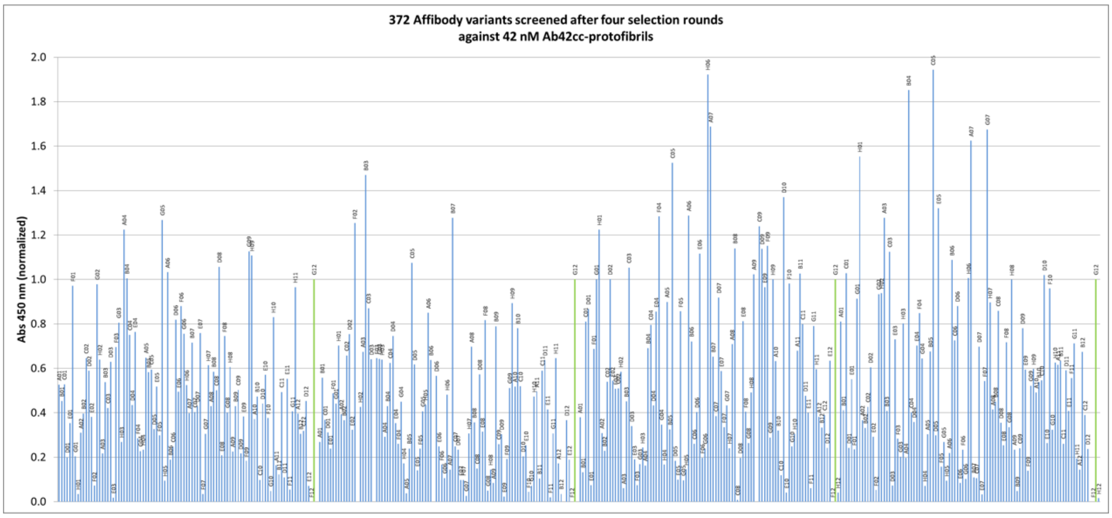

b.

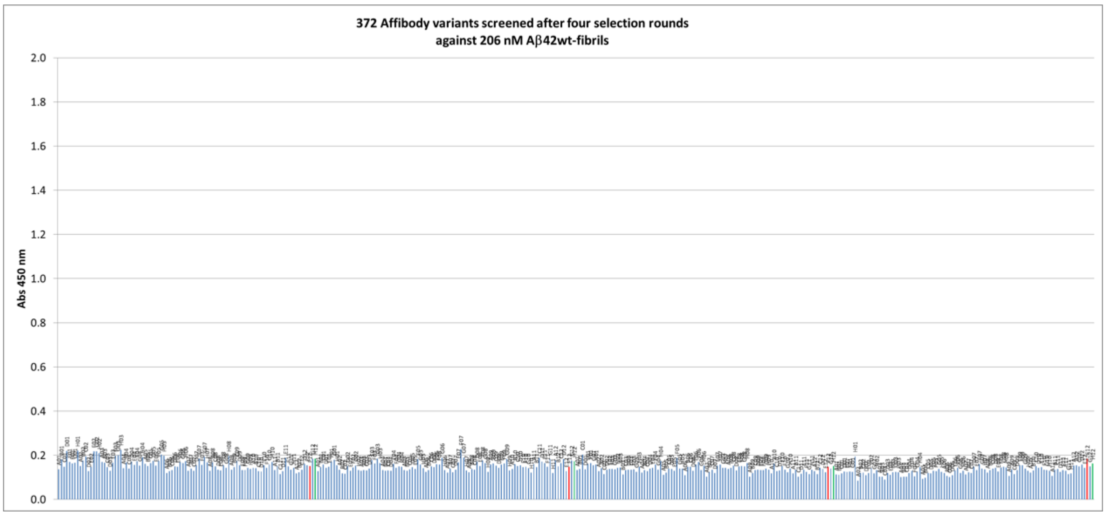

Figure S1. (**a**) Single-point ELISA screen for $A\beta_{42}$cc protofibril binding by 372 Affibody variants collected after four rounds of selections. (**b**) A corresponding comparison of binding to amyloid fibrils of wild type $A\beta_{42}$. The $A\beta_{42}$cc protofibril concentration was 42 nM (200 ng/mL) and $A\beta_{42}$wt fibrils 206 nM (950 ng/ml) based on monomer concentration.

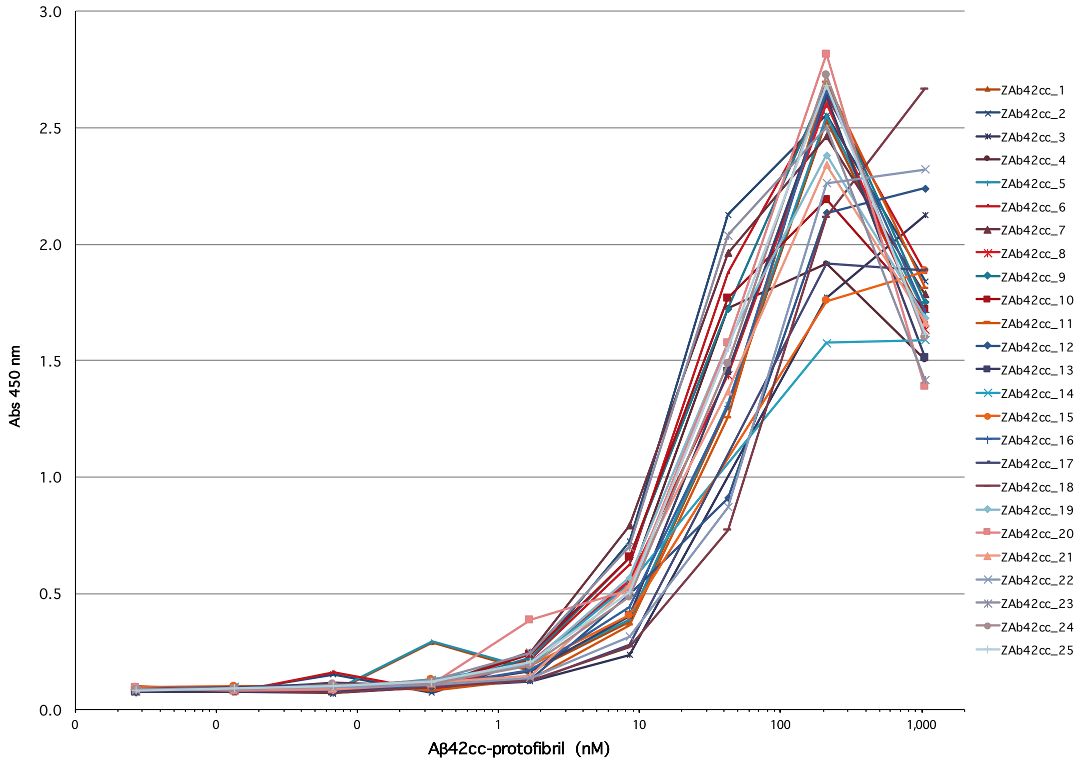

Figure S2. EC_{50}-ELISA of the 25 best performing Affibody molecules assayed for binding of $A\beta_{42}$cc-protofibrils (1,000-0.01 nM). All Affibody molecules show a concentration dependent binding response to $A\beta_{42}$cc-protofibrils. Estimated EC_{50} values (in this ELISA format) are in the range of 15 to 90 nM for the best 25 binders.

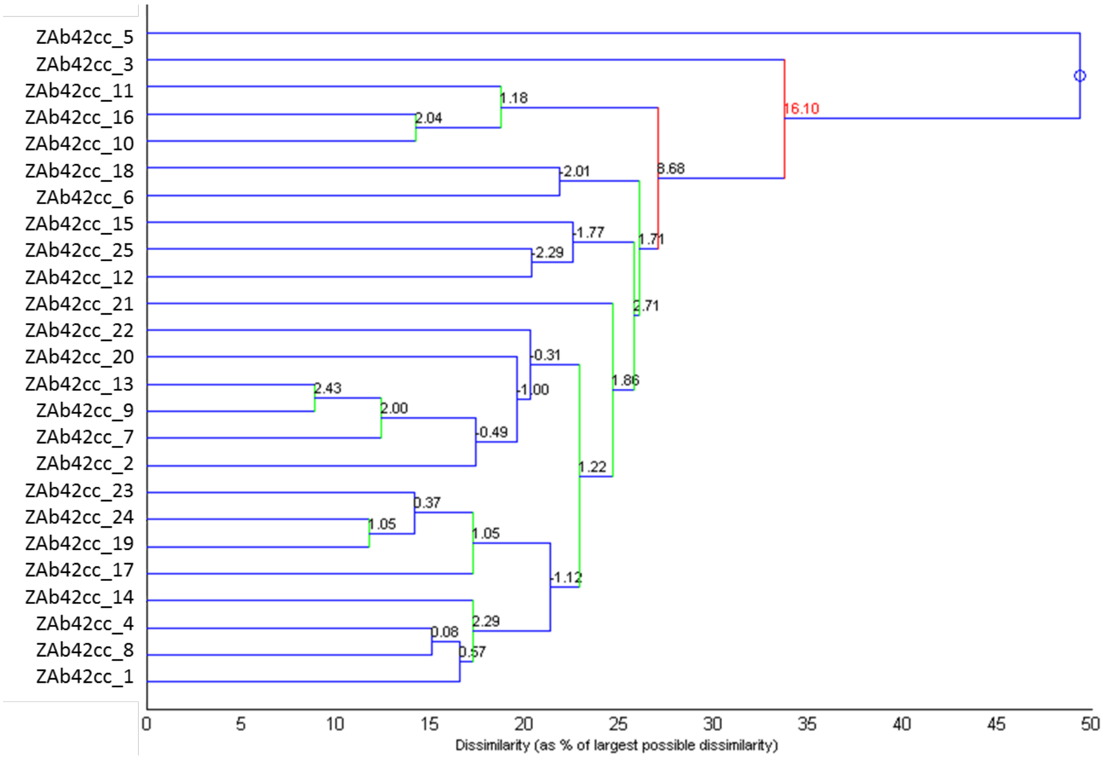

Figure S3. Sequence comparison (phylogenetic tree) of 25 selected Affibody molecules.

Table S1. Equilibrium dissociation constant (K_D), association rate constant (k_a), and dissociation rate constant (k_d) for the Affibody molecules to $A\beta_{42}cc$ protofibrils.

Affibody molecule	K_{D1} (nM, mean ± SD)	k_{a1} ($M^{-1}s^{-1}$, mean)	k_{d1} (s^{-1}, mean)	K_{D2} (nM, mean ± SD)	k_{a2} ($M^{-1}s^{-1}$, mean)	k_{d2} (s^{-1}, mean)
$ZA\beta_{42}cc\_1$	1.6 ± 0.06	2.9×10^5	4.7×10^{-4}	7.0 ± 0.49	1.2×10^7	8.6×10^{-2}
$ZA\beta_{42}cc\_2$	1.1 ± 0.02	7.2×10^5	7.3×10^{-4}	9.7 ± 0.02	5.8×10^6	5.6×10^{-2}
$ZA\beta_{42}cc\_4$	1.4 ± 0.01	2.5×10^5	6.3×10^{-4}	8.7 ± 0.54	3.4×10^6	3.0×10^{-2}
$ZA\beta_{42}cc\_5$	2.5 ± 0.14	3.5×10^5	4.8×10^{-4}	15.6 ± 0.10	1.3×10^{10}	2.0×10^2
$ZA\beta_{42}cc\_4$-$ZA\beta_{42}cc\_4$	0.3 ± 0.00	3.4×10^5	9.2×10^{-5}	5.3 ± 0.02	2.4×10^{10}	1.3×10^2
$ZA\beta_{42}cc\_4$-(G_3S)-$ZA\beta_{42}cc\_4$	0.6 ± 0.01	3.4×10^5	1.9×10^{-4}	4.4 ± 1.40	1.8×10^{10}	8.2×10^1
$ZA\beta_{42}cc\_4$-$(G_3S)_2$-$ZA\beta_{42}cc\_4$	0.7 ± 0.01	3.2×10^5	2.4×10^{-4}	2.7 ± 0.01	1.1×10^{11}	3.1×10^2
$ZA\beta_{42}cc\_4$-$(G_3S)_3$-$ZA\beta_{42}cc\_4$	0.6 ± 0.01	4.0×10^5	2.4×10^{-4}	4.5 ± 0.02	7.3×10^{10}	3.4×10^2
$ZA\beta_{42}cc\_4$-$(G_3S)_4$-$ZA\beta_{42}cc\_4$	1.1 ± 0.05	2.2×10^5	2.5×10^{-4}	3.8 ± 3.80	1.3×10^9	4.9
$ZA\beta_{42}cc\_1$-ABD	2.3 ± 0.01	2.0×10^5	4.8×10^{-4}	19.3 ± 0.19	3.2×10^5	6.2×10^{-3}
$ZA\beta_{42}cc\_1$-(G_4S)-$ZA\beta_{42}cc\_1$-ABD	1.7 ± 0.02	3.1×10^5	5.2×10^{-4}	5.2 ± 0.14	2.5×10^7	1.4×10^{-1}
$ZA\beta_{42}cc\_1$-$(G_4S)_2$-$ZA\beta_{42}cc\_1$-ABD	n.d.	6.2×10^4	n.d.	2.6 ± 0.08	1.4×10^7	3.6×10^{-2}
$ZA\beta_{42}cc\_1$-$(G_4S)_4$-$ZA\beta_{42}cc\_1$-ABD	1.1 ± 0.03	1.4×10^5	1.6×10^{-4}	8.4 ± 0.46	1.5×10^9	1.2×10^1
$ZA\beta_{42}cc\_3$-ABD	0.9 ± 0.02	1.3×10^5	1.3×10^{-4}	4.6 ± 0.13	6.6×10^5	3.1×10^{-3}
$ZA\beta_{42}cc\_3$-(G_4S)-$ZA\beta_{42}cc\_3$-ABD	0.2 ± 0.01	1.3×10^5	2.1×10^{-5}	56.2 ± 5.50	7.1×10^4	4.0×10^{-3}
$ZA\beta_{42}cc\_3$-$(G_4S)_2$-$ZA\beta_{42}cc\_3$-ABD	0.9 ± 0.03	3.9×10^4	3.7×10^{-5}	7.1 ± 0.17	3.3×10^5	2.3×10^{-3}
$ZA\beta_{42}cc\_3$-$(G_4S)_4$-$ZA\beta_{42}cc\_3$-ABD	0.1 ± 0.01	1.5×10^5	1.6×10^{-5}	17.2 ± 2.40	1.4×10^5	2.5×10^{-3}
$ZA\beta_{42}cc\_5$-ABD	1.9 ± 0.07	3.1×10^5	6.2×10^{-4}	24.6 ± 1.20	3.2×10^7	8.0×10^{-1}
$ZA\beta_{42}cc\_5$-(G_4S)-$ZA\beta_{42}cc\_5$-ABD	0.1 ± 0.00	3.1×10^6	3.7×10^{-4}	14.8 ± 0.50	6.6×10^6	9.8×10^{-2}
$ZA\beta_{42}cc\_5$-$(G_4S)_2$-$ZA\beta_{42}cc\_5$-ABD	2.0 ± 0.03	2.5×10^5	5.2×10^{-4}	17.5 ± 0.30	1.9×10^6	3.3×10^{-2}
$ZA\beta_{42}cc\_5$-$(G_4S)4$-$ZA\beta_{42}cc\_5$-ABD	2.8 ± 0.04	1.6×10^5	4.5×10^{-4}	17.2 ± 0.26	1.3×10^6	2.3×10^{-2}
